# Temperature Influence on Formability and Microstructure of AZ31B during Electric Hot Temperature-Controlled Incremental Forming

**DOI:** 10.3390/ma14040810

**Published:** 2021-02-08

**Authors:** Haoran Zhang, Xingrong Chu, Shuxia Lin, Huawei Bai, Jiao Sun

**Affiliations:** Associated Engineering Research Center of Mechanics and Mechatronic Equipment, Shandong University, Weihai 264209, China; zhanghaoran_sxty@163.com (H.Z.); linshuxia@sdu.edu.cn (S.L.); 18369905057@163.com (H.B.); sunjiao1980@126.com (J.S.)

**Keywords:** Magnesium alloy sheet, electric, incremental forming, microstructure, deformation behavior

## Abstract

The straight groove test of AZ31B magnesium alloy sheet by electric hot temperature-controlled incremental sheet forming (ISF) was conducted at different temperatures. The temperature influence on fracture depth, deformation force and strain distribution was investigated. It was found that the limit depth and major strain increased as the temperature rose and that the forming force decreased correspondingly. Furthermore, the fracture behavior changed from brittle fracture to ductile fracture. Considering the formability and surface wear comprehensively, the optimized forming temperature was determined to be 300 °C. The microstructure of the groove specimen was analyzed and the dynamic recrystallization (DRX) was considered to be the reason for the improved formability. The degree of DRX depended on the temperature and degree of deformation, which resulted in non-uniform distribution of hardness within the cross section of the groove specimen.

## 1. Introduction

On account of the characteristics of low density and a high ratio of strength to density, magnesium alloys have been widely used in the fields of aerospace, transportation and electronics. However, magnesium alloys present poor plasticity at room temperature as a result of their hexagonal close-packed crystal structure [1,2]. As an unconventional sheet forming technology, incremental sheet forming (ISF) technology can improve the forming limit of the sheet by replacing the traditional whole forming with successive partial forming [3,4,5].

Warm incremental sheet forming (WISF) was developed and applied to magnesium alloy sheet forming on the basis of ISF. Zhang et al. [6] studied the effect of anisotropy on the incremental forming properties of AZ31 sheet by furnace heating and proposed that the cross-rolling sheet was more suitable for the WISF process. Ambrogio et al. [7] indicated that the maximum formability of AZ31 sheet was obtained at 200–300 °C with the whole uniform heating method. Xu et al. [8] confirmed the advantages of friction–agitation ISF with AZ31 plates—which presented the highest heating efficiency at the rotational speed of 5000 rpm. Zhang et al. [9] heated the AZ31 sheet globally through an oil bath and found that the most influential factor affecting formability was the forming temperature, followed by step depth, sheet thickness and tool diameter.

Considering the high efficiency of electric heating, electrically assisted processing is promising to achieve increased deformation for magnesium alloys [10,11]. Scholars have carried out related research on electro-assisted hot incremental forming (EHIF). Xu et al. [8] pointed out that the method of using current to realize local heating had advantages in heating efficiency. Fan et al. [12] carried out an electric heating incremental forming of magnesium alloy and the forming limit angle reached the maximum value with a current of 500 A, a feed rate of 1000 mm/min, a tool diameter of 8 mm and a step size of 0.2 mm. Xu et al. [13] applied electric pulses to the double-side multi-point incremental sheet forming (DSIF) and developed a hybrid DSIF toolpath strategy to eliminate the geometrical deviation that is due to bending. Van Sy et al. [14] made the electric current pass through the AZ31 sheet to achieve overall heating during the ISF process, which resulted in the improvement of accuracy and formability. Bao et al. [15] applied a constant pulse current to AZ31 sheet and confirmed that electric heating reduced the dynamic recrystallization (DRX) temperature of magnesium alloy in the ISF process when compared with traditional heating.

In this work, based on a self-made experiment platform, a method to control the tool forming temperature was proposed. The formability of AZ31B sheet at different temperatures was studied by means of the straight groove test, which was used by Kim et al. [16] to evaluate forming limit curves in ISF. The fracture depth, strain distribution and multiple forces in ISF at different forming temperatures was obtained. In addition, the effect of temperature on the microstructure evolution of different areas and fracture features was investigated in detail.

## 2. Experimental Procedure

**Figure 1:**
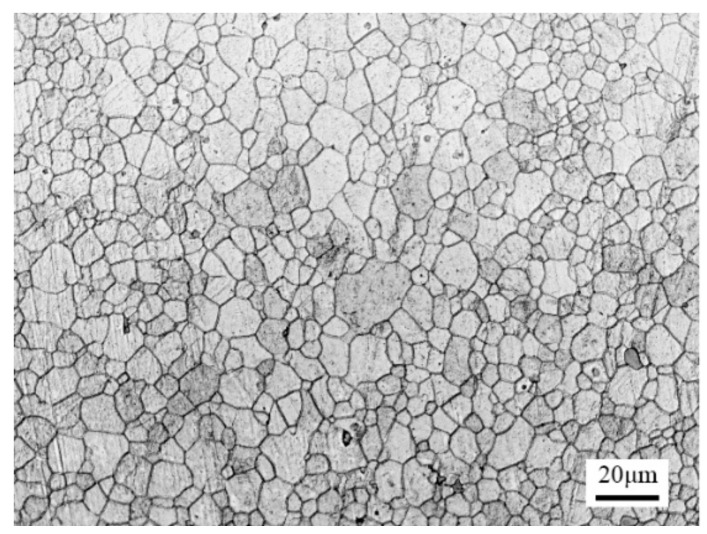
Microstructure of as-received AZ31B alloy.

The material used in this article was AZ31B alloy sheets (1 mm thick). The chemical composition (wt.%) of the material was: 2.99% Al, 0.64% Zn, 0.34% Mn, 0.16% Ca, 0.08% Si, 0.15–0.35% Cr,0.1% Zr and the balance of magnesium. The as-received microstructure is shown in Figure 1. Most of the grains were uniform and equiaxed. The sheet was cut into rectangles with dimensions of 70 mm × 100 mm for the groove test.

**Figure 2:**
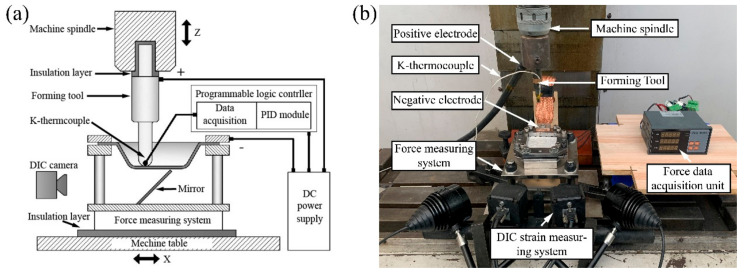
Experimental equipment of EHIF: (**a**) schematic structure, (**b**) actual setup.

The schematic structure and actual composition of the self-made experimental platform are shown in Figure 2. The deformation system was built on the traditional vertical three-axis milling machine (Model XKA714, Beijing, China). The DC power supply used in the test could output voltage of 15 V and current of 0–1500 A, and its positive and negative poles were connected to the tool and magnesium alloy plate, respectively. The current passed through the tool and the plate to achieve local heating. The sampling point of the proportion integration differentiation (PID) controller feedback signal was set on the top of the tool and as close as possible to the contact position between the tool and the plate without disturbing the forming process. The current output of the power supply was adjusted to maintain the stability of the temperature with the tool temperature, obtained by the K-thermocouple as a reference. Therefore, the tool temperature remained stable throughout the process and, correspondingly, the plate temperature was indirectly controlled. In addition, the temperature of the plate was obtained by multi-channel temperature measurement (Module SH-X). By setting fixed sampling points on the back of the magnesium alloy plate, the actual temperature variation trend on the plate was obtained.

**Figure 3:**
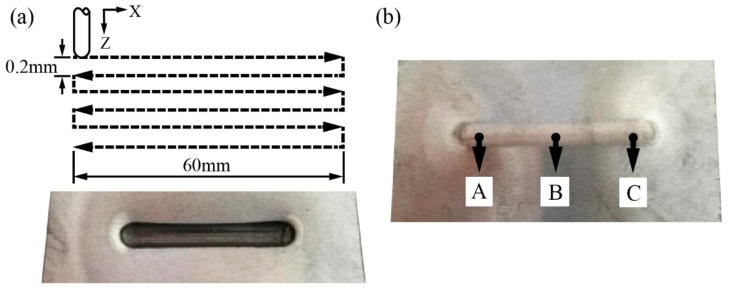
(**a**) Schematic diagram of the tool path for forming a straight groove and (**b**) sampling points A, B and C on the back of the plate.

The tool path was controlled by the NC program in the FANUC system as shown in Figure 3a. The horizontal and vertical feeds of the machine corresponded to the X and Z directions, respectively. The length of the straight groove was 60 mm and the processing parameters were a step size of 0.2 m, a feed rate of 500 mm/min and a tool diameter of 8 mm. The target temperatures of the tool were set to 100 °C, 150 °C, 200 °C, 250 °C, 300 °C and 350 °C. The experiment was repeated three times under each condition. In order to study the temperature variation during the machining path, the actual temperature of the plate was measured at point A, B and C, as shown in Figure 3b, to compare with the target tool temperatures. Based on the digital image correlation (DIC) strain measuring system (Module XJTUDIC, Xi’an, China), the strain distribution of groove specimens was obtained. The forming force data were acquired by the measuring system, which consisted of the data acquisition card and the personal computer (PC). The mixture of MoS_2_ power and acetone solution was applied as the lubricant to reduce the friction between the tool and sheet. The microstructure was observed by the optical microscope (Module Axio Lab A, Jena, Germany) and the fracture behavior was analyzed by the scanning electron microscope (SEM, Module Nova Nano SEM 450, Hillsboro, OR, USA). Microhardness measurement was carried out with the Vickers hardness testing machine (ModuleMHV-100Z LCD, Beijing, China). The load used was 0.1 kg and the holding time was 10 s.

## 3. Results and Discussions

### 3.1. Temperature Difference

**Figure 4:**
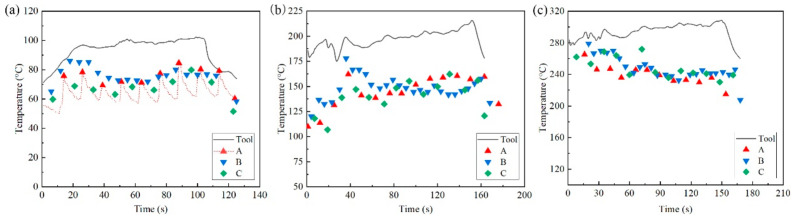
Real-time temperature of the tool head and sampling points A, B and C: (**a**) target temperature 100 °C, (**b**) target temperature 200 °C and (**c**) target temperature 300 °C.

Figure 4 shows the actual tool temperature and the temperature of the three positions A, B and C on the sheet when the target tool temperature was set to 100 °C, 200 °C and 300 °C. At the beginning of the experiment, the tool temperature rose rapidly to the target temperature and then gradually stabilized. Then, the temperature dropped rapidly after the power supply stopped output. As the tool moved back and forth and periodically passed through the points A, B and C, the temperature of the fixed point on the plate fluctuated with time, as shown in Figure 4a. The peak value corresponded to the tool approaching the measurement point and the valley value corresponded to the moment when the tool was furthest from the sampling point. Therefore, the peak temperature was regarded as the actual temperature on the sheet under temperature control conditions, as shown in Figure 4a.

As shown in Figure 4, there was a difference between the real-time temperature of the plate and that of the tool during the forming process. Referring to the research of Ao et al. [17] and Xu et al. [8] on the electric heating in EHIF, the Joule heat generation in this experiment followed the relationship in Equation (1).

(1) $$Q=I^{2}(k\gamma_{t}+\gamma_{c}+\gamma_{s})t$$

where *Q* is the thermal energy generated in the local forming area, *I* is the current density, k is the coefficient of heat conduction, *γ_t_* is the electrical resistivity of the tool, *γ_c_* is the contact electrical resistivity between the tool and the sheet, *γ_s_* is the electrical resistivity of AZ31B sheet and t is the forming time. This equation is composed of three main parts and was only satisfied at the position where the tool was in direct contact with the plate. As a result, it could be inferred that the temperature of the contact position was the highest near the sampling position of the K-type thermocouple on the tool head. It was easier to dissipate heat from the back of the sheet. Therefore, the actual temperature measured at points A, B and C was lower than the actual temperature of the tool head. As shown in Figure 4, when the target temperature of the tool was set to 100 °C, 200 °C and 300 °C, the average temperature of the three sampling points on the plate was approximately maintained at 73 °C, 145 °C and 240 °C, respectively. The temperature difference tended to increase as the controlled temperature increased. 

### 3.2. Formability

#### 3.2.1. Fracture Depth

**Figure 5:**
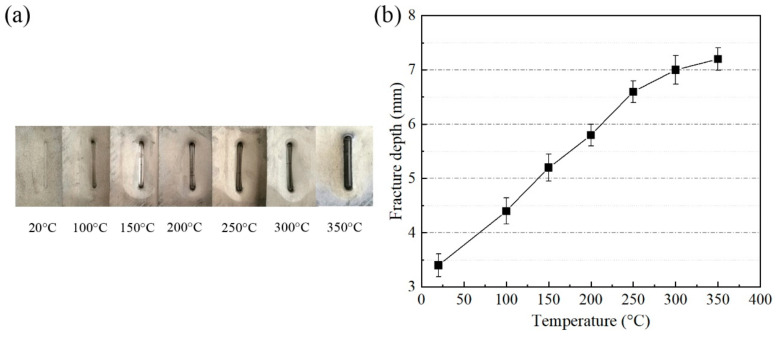
(**a**) Groove specimens and (**b**) fracture depth corresponding to different temperatures.

The groove specimens and the corresponding fracture depth under different tool temperatures are shown in Figure 5. The fracture depth was 3.4 mm at room temperature (20 °C) and it was increased to 7.2 mm at 300 °C, presenting an increase of 112%. It was shown that the fracture depth linearly increased with temperature from 20 °C to 300 °C. At 350 °C, the fracture depth of the groove was 7.4 mm, an enhancement of only 0.2 mm compared with 300 °C. However, the surface oxidation and abrasion of the grooved specimen were obvious at 350 °C. Considering the wear protection of the tool and the surface quality of the specimen, 300 °C was selected as the optimized target temperature. The groove parts that formed between 20 °C and 300 °C were selected as the analysis specimens.

#### 3.2.2. Deformation Force

**Figure 6:**
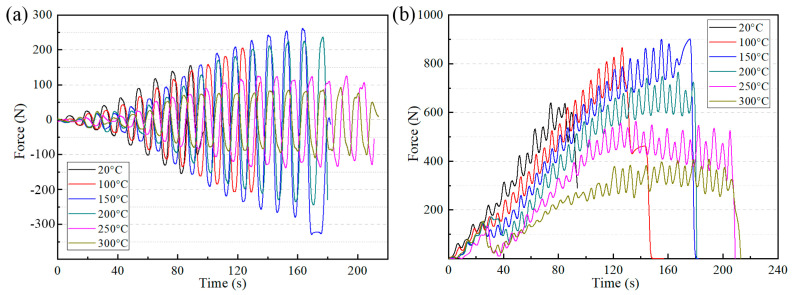
(**a**) Horizontal forming forces and (**b**) vertical forming forces.

The horizontal and vertical forming forces at different temperatures are shown in Figure 6. For the characteristics of the groove experiment, the deformation force perpendicular to the *Y*-axis direction was basically zero. Hence, only the force in the X direction is discussed. As shown in Figure 6a, the direction of horizontal force changed alternately between positive and negative due to the tool head moving back and forth. When the temperature was lower than 250 °C, the deformation force increased continuously, regardless of whether it was horizontal or vertical. Work hardening caused by material deformation was the main reason for this phenomenon. When the temperature was above 250 °C, the maximum horizontal force reduced markedly and remained stable from the middle stage of forming. The same trend was observed on the force curve of the vertical direction. This indicated that the hardening and softening effects reached a balance between each other above 250 °C. When the target temperature was set to 300 °C, the vertical and horizontal forming force decreased obviously. The above indicates that the forming temperature had a significant effect on the forming force in EHIF and that the deformation force decreased with increasing temperature.

#### 3.2.3. Strain Distribution

**Figure 7:**
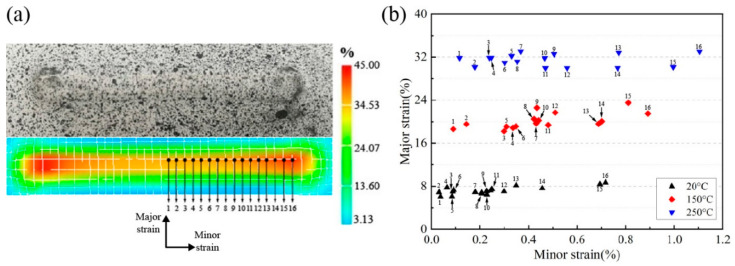
(**a**) Equivalent strain cloud chart and (**b**) major strain and minor strain along the groove.

In this part, the DIC system was used to analyze the strain distribution of groove specimens. Figure 7a presents the plane strain of the groove specimen at the moment of sheet fracture at room temperature, 150 °C and 250 °C. The major strain and minor strain of the 16 points marked in the middle area along the groove direction were collected as shown in Figure 7b. The major strain was perpendicular to the groove direction whilst the minor strain was along the groove direction. The major strain of each sampling point was similar. However, the minor strain at the point near the end of the straight groove was maximum. This is because the deformation at the end points showed a characteristic towards the biaxial strain rather than the plane strain [17,18]. At room temperature, the major strain was mainly distributed in range from 4% to 10%. With increasing temperature, the major strain was increased to 17–24% at 150 °C and 28–36% at 250 °C. This indicated that as the target temperature rose, the major and minor strains of the material in the forming area tended to increase, which meant that the forming limit was improved.

**Figure 8:**
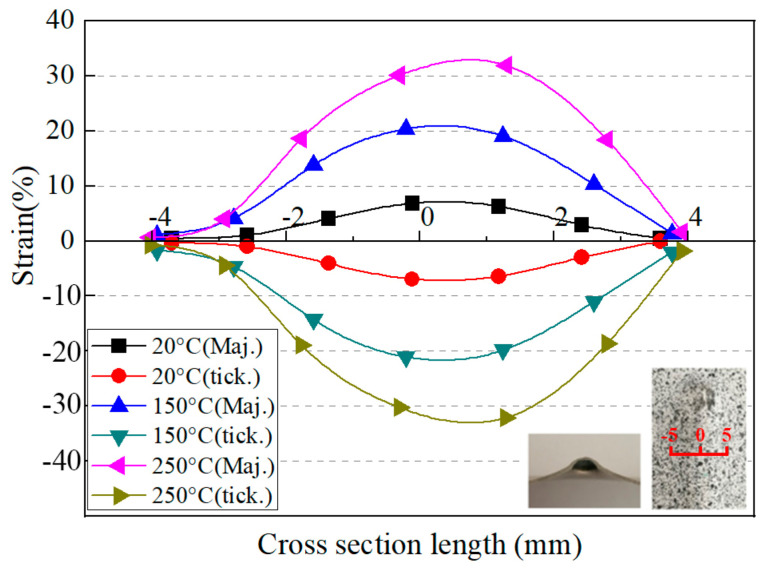
Major strain and thickness strain map along the investigated cross section.

According to the law of volume invariance during the plastic deformation process, the thickness strain of the sheet could be obtained by the DIC system. Figure 8 shows the temperature influence on the major strain and thickness strain in the cross section. The selected temperatures were 20 °C, 150 °C and 250 °C. The thickness reduction was the largest in the middle region of the cross section at the same temperature and, correspondingly, the major strain was the largest. The thickness reduction was 7% at 20 °C and it was improved to 22% at 150 °C and 33% at 250 °C. The higher thickness strain presented the improvement of sheet formability. As shown in Figure 7 and Figure 8, the minor strain, the major strain and the thickness strain showed the same distribution characteristics at different temperatures. They all increased with the increasing temperature. This was consistent with the change in formability presented in Figure 5. Due to the elevated temperature and large deformation, the paint spots on the formed specimen easily fell off, resulting in the failure of the DIC strain analysis. Hence, the strain distribution at 300 °C was not included in this part.

### 3.3. Microstructure Evolution

**Figure 9:**
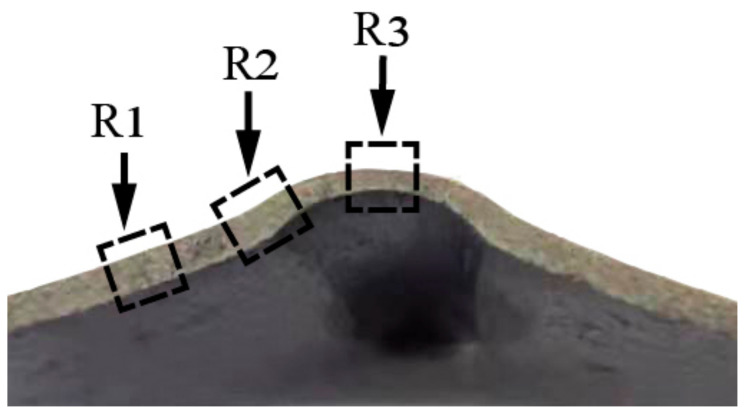
Sketch of specimen positions in the transverse section.

**Figure 10:**
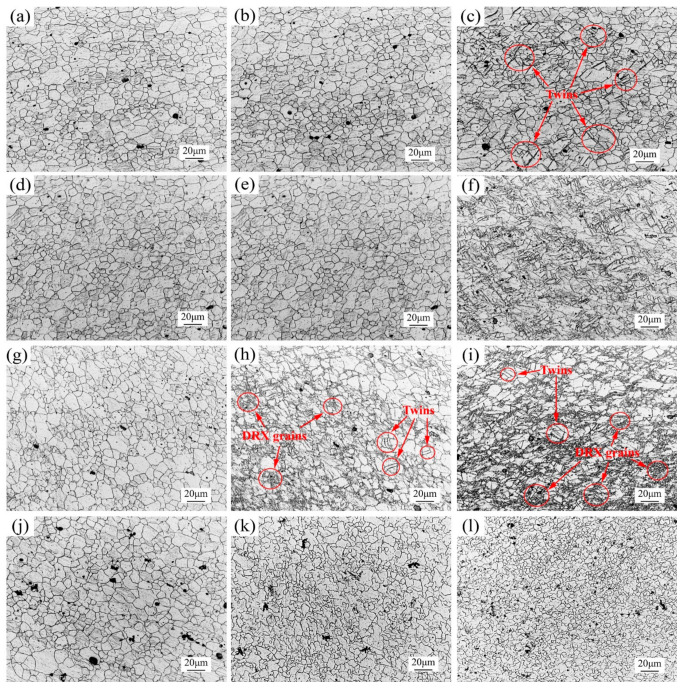
Microstructures in the transverse section of different regions at different temperatures: (**a**–**c**) R1–R3 zone at 20 °C, (**d**–**f**) R1–R3 zone at 150 °C, (**g**–**i**) R1–R3 zone at 250 °C and (**j**–**l**) R1–R3 zone at 300 °C.

Figure 9 presents the metallographic sampling locations marked with the bending forming region (R1), the middle forming region (R2) and the final forming region (R3), respectively. The microstructure evolution of groove specimens deformed at 20 °C, 150 °C, 250 °C and 300 °C in the cross section is shown in Figure 10.

The microstructures of groove specimens deformed at 20 °C are shown in Figure 10a–c. Figure 10a presents the equiaxed grain structure of the R1 zone, which was similar to the as-received microstructure. Figure 10a also shows that some twins occurred in the R2 zone when compared with the R1 zone. It was explained that the non-basal slip system of magnesium alloy was difficult to be activated and twinning was the main deformation mechanism at room temperature [19,20]. As shown in Figure 10c, the grains became elongated slightly and more twins were obtained for greater deformation in the final forming zone R3. Figure 10d–f show the microstructure evolution of specimens deformed at 150 °C. This is because the EHIF process achieved local heating and had little effect on the bending forming area. The microstructure characteristics in Figure 10d,e were similar to the microstructure in Figure 10a,b. Compared with Figure 10e, more twins and fine grains were observed in the final forming zone, as shown in Figure 10f. Plastic deformation most easily occurred under the combined effect of twinning and dynamic recrystallization for magnesium alloy [21]. Twins could promote the migration of new high-angle grain boundaries and the nucleation of grain protrusion [22]. When the temperature was controlled at 150 °C twinning and DRX worked together, which was the main reason for the improvement in sheet formability.

The microstructures of groove specimens deformed at 250 °C are shown in Figure 10g–i. As seen in Figure 10g, the microstructure was also similar to the as-received microstructure. However, twins almost disappeared and the grain boundary became more non-uniform in the middle forming zone. Some finer grains appeared at the intersection triangle of partial grain boundaries. DRX became the main deformation coordination mechanism. This was because the temperature condition for DRX of magnesium alloy (200 °C) was reached [23] and greater deformation was obtained compared with other temperatures. As shown in Figure 10i, the degree of dynamic recrystallization increased obviously. In Figure 10k,l, the irregular grain boundaries and significantly reduced grain size indicated that new DRX grains have grown and that the degree at 300 °C was the highest among all temperatures. This corresponded to the result that the macro forming force no longer increased during the forming process but decreased instead.

### 3.4. Fracture Characteristic

**Figure 11:**
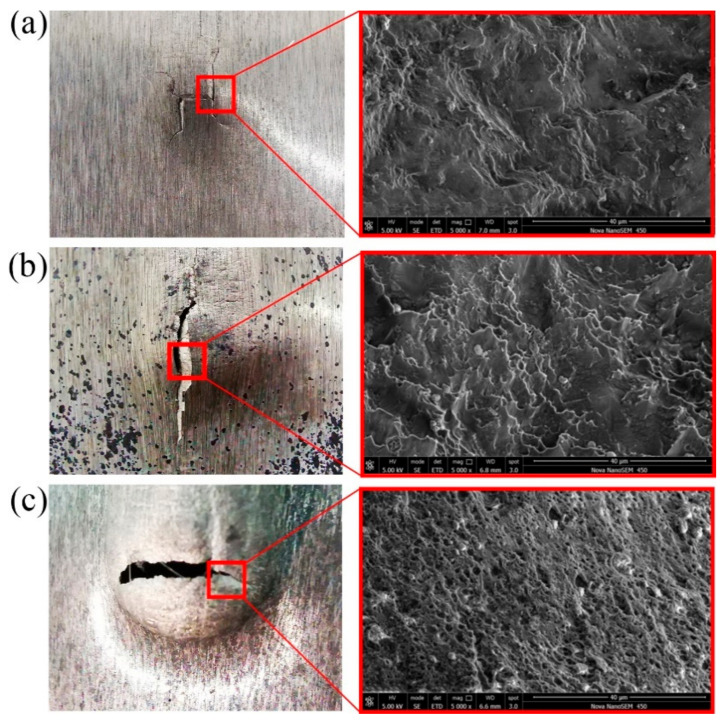
Comparison of SEM fracture graphs: (**a**) 20 °C, (**b**) 150 °C and (**c**) 250 °C.

The fracture of the sample observed at 20 °C, 150 °C and 250 °C is shown in Figure 11. The initial fracture was located at the end of the groove due to the larger strain. Figure 11a consisted of the cleavage platform and the tearing edge. The typical brittle fracture for the specimens was observed at room temperature. When the temperature increased to 150 °C, the cleavage platform also existed and the scale of the tearing edge became larger. Meanwhile, the fracture morphology contained some shallow dimples, showing the characteristics of plastic fracture [24]. Figure 11c shows that the fracture surface was covered with a number of small dimples with a ductile fracture feature. This proved that the sheet forming performance was significantly improved when the temperature was set to 250 °C.

### 3.5. Microhardness

**Figure 12:**
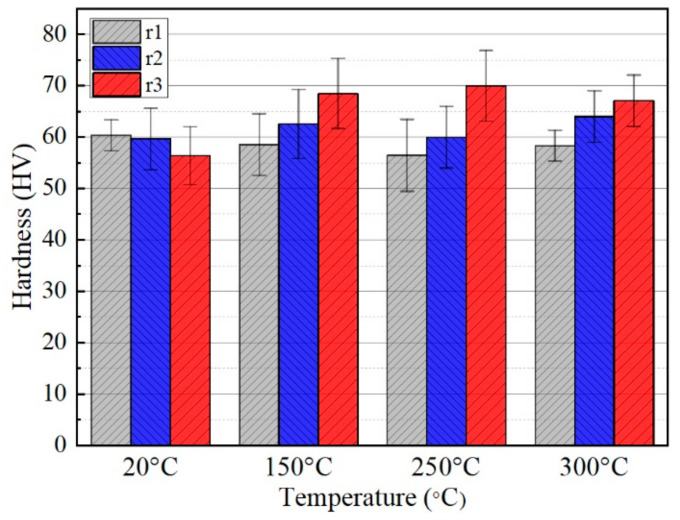
Average microhardness of specimens in the transverse section of different regions at different temperatures.

The microhardness of the sample in different regions (the R1, R2 and R3 zones in Figure 9) and at 20 °C, 150 °C, 250 °C and 300 °C is shown in Figure 12. At room temperature, coarse grains remained after deformation due to the limited deformation, as shown in Figure 10a–c. Hence, the microhardness of the three regions changed insignificantly. When the temperature rises to 150 °C, the hardness of the deformation zones was different and the final forming zone presented the largest value. The same increasing trend could also be found at 250 °C and 300 °C. DRX significantly refined grains, as shown in Figure 10h,i and Figure 10k,l. As DRX led to the increase in the degree of grain refinement, the strengthening of grain boundaries became more and more obvious [25]. This caused an improvement in microhardness from region R1 to R3. Although the degree of DRX was the highest in the final forming area at 300 °C, the new crystal grains had grown and the hardness had not been significantly improved.

## 4. Conclusions

The straight groove test was carried out based on a self-built EHIF platform. The real-time temperature of the tool was used as a feedback signal to control the forming temperature. Furthermore, the actual temperature of the sheet was measured. The temperature influence on the formability of AZ31 sheets was studied from the aspects of fracture depth, deformation force and strain distribution. The conclusions obtained are as follows:

1. The optimized controlled forming temperature of the AZ31B EHIF process was 300 °C. The fracture depth increased from 3.4 mm to 7.2 mm, which presented an increase of 112% compared with the traditional ISF at room temperature.
2. The deformation force decreased and the major strain increased with the increasing temperature, which proved the formability enhancement of AZ31B sheet. The forming force dropped significantly and remained stable when the set temperature was higher than 250 °C.
3. The temperature presented the greatest influence on the microstructure of the final forming zone when compared with the bending forming zone and the middle forming zone. DRX became the main deformation coordination mechanism above 250 °C and significantly improved the formability of AZ31B sheet.
4. The fracture behavior of specimens changed from the typical brittle fracture to the ductile fracture from 20 °C to 250 °C due to the improvement in plasticity.

## Data Availability

The processed data required to reproduce these findings cannot be shared at this time as the data also forms part of an ongoing study.
